# Protective effect of TSLP delivered at the gut mucosa level by recombinant lactic acid bacteria in DSS-induced colitis mouse model

**DOI:** 10.1186/s12934-015-0367-5

**Published:** 2015-11-06

**Authors:** Camille Aubry, Christophe Michon, Florian Chain, Yolande Chvatchenko, Laurence Goffin, Simone C. Zimmerli, Sylvia Leguin, Philippe Langella, Luis Bermudez-Humaran, Jean-Marc Chatel

**Affiliations:** INRA, UMR1319 Micalis, 78350 Jouy-en-Josas, France; AgroParisTech, UMR Micalis, 78350 Jouy-en-Josas, France; Merck Serono SA, 1170 Aubonne, Switzerland; EMD Serono, 45A Middlesex Turnpike, Billerica, MA 01821 USA

**Keywords:** TSLP, Mucosal delivery, Inflammatory bowel disease, *Lactococcus lactis*

## Abstract

**Background:**

Thymic stromal lymphopoietin (TSLP) is a cytokine known to mature dendritics cells, lower pro-inflammatory IL-12 secretion, induce differentiation of anti-inflammatory FoxP3+ regulatory T cells (Treg). Moreover, Crohn’s disease patients have shown a reduction of intestinal TSLP expression. To understand the role of TSLP in inflammation, we constructed *Lactococcus lactis* strain producing TSLP (LL-TSLP) and investigated the effect of its administration on dextran sulfate sodium (DSS)-induced colitis model in mice.

**Results:**

LL-TSLP secrete an active molecule which lowers secretion of IL-12 by dendritic cells. Treatment with LL-TSLP, increases the amount of TGF-β secreted by T cells in Mesenteric Lymph Node in healthy mice. In acute DSS-induced colitis, LL-TSLP delayed the Disease Activity Index and lowered histological score and colonic INF-γ production. In a DSS-recovery model, LL-TSLP induced a better protective effect if the strain was administered at the beginning of the colitis. At Day 4 of colitis we observed an induction of Treg by LL-TSLP.

**Conclusions:**

TSLP showed an anti-inflammatory protective role in DSS-induced colitis. We have demonstrated that a short and early administration of LL-TSLP is more efficient than a long lasting treatment.

## Background

TSLP was first discovered in a thymic stromal cell line [1]. It is mainly produced by non-hematopoietic cells as keratinocytes and epithelial cells [2–4] in response to stress stimuli [5, 6]. TSLP is a cytokine well known in the homeostasis of TH2 immune response [5]. It has been implied in several diseases like asthma, allergic rhinitis, food allergy [7–9] but also in cancer [10, 11]. However despite its key role in allergic response, TSLP has been shown to have protecting effects in a mouse model of colitis [4, 12, 13]. Indeed, TSLP is implied in the differentiation of CD4+CD25-thymocyte into FoxP3+ regulatory T cells (Treg) by the intermediate of human myeloid Dendritic Cells (DCs) or human plasmocyte DCs [14, 15]. However, in mice, TSLP doesn’t affect DCs directly but is secreted by those cells and allows the promotion of Treg [13]. TSLP also acts on human and murine DCs by inhibiting IL-12 secretion which is a pro-inflammatory protein involved in Inflammatory Bowel Disease (IBD) [3, 4].

IBD which gathers Crohn’s disease (CD) and Ulcerative Colitis (UC) affects 1.4 million Americans and the prevalence rate is 396 per 100,000 individuals worldwide [16]. Incidence and prevalence are increasing in various regions of the world including the ones which were less impacted [17, 18]. Due to its symptoms (diarrhea, abdominal pain, loss of weigh) IBD is considered as an incapacitating disease. Patients have a higher risk factor to develop other inflammatory or non-inflammatory disorders like psoriasis, cancer or arthritis [19–22]. So far no curative treatments exist for the disease. The most powerful treatment is the injection of the recombinant antibodies targeting TNF-α, however even if 60 % are primary responders this drops to 25–40 % still in remission after 1 year of treatment [23]. The last solution in IBD is surgery where inflamed parts of the intestine are withdrawn. However surgery can lead to severe complications as Short Bowel syndrome and relapses are frequent. All together, this makes IBD one of the major health problems in developed country and the development of innovative therapeutics or curative strategies is crucial.

One of the ways explored to help in alleviating symptoms of the disease is the delivery of anti-inflammatory molecules by recombinant lactic acid bacteria (LAB). Recently, it has been shown that mice treated with LAB expressing the protease inhibitor Elafin were protected against gut inflammation [24]. LAB have been used for 1000 years for food conservation and appear to be a promising vehicle delivering active molecules [25]. They are recognized as safe by World Health Organization, and some strains can have anti-inflammatory properties [26].

In this study we decided to construct *Lactococcus lactis* expressing TSLP (LL-TSLP) in order to study the effect of a local gut mucosal administration of TSLP on DSS-induced inflammation. LL-TSLP constructs demonstrated anti-inflammatory properties in vitro and protected mice from DSS-induced colitis after oral administration as shown by reduced weight loss, lower disease activity and microscopic score. Moreover mice were protected even if they were fed with LL-TSLP only during the four first days of the colitis. Additionally we showed that part of this protective effect was due to a higher recruitment of Treg.

## Results

### Secretion of biologically active TSLP by a recombinant *Lactococcus lactis* strain

**Fig. 1:**
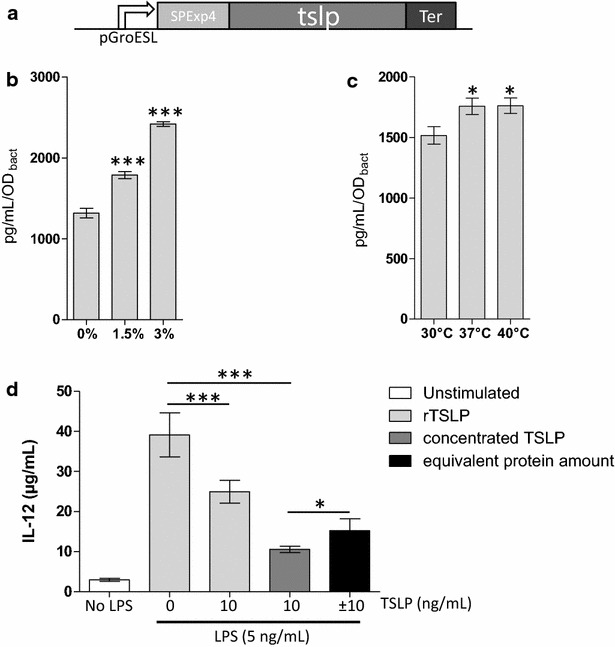
Secretion of biologically active TSLP by recombinant *Lactococcus lactis.* **a** Schematic representation of the expression cassette: GroESL promoter (pGroESL) followed by *tslp* gene, flanked by the signal peptide (SP) of Exp4 and a terminator (Ter). **b** Detection of TSLP by ELISA in supernatant fractions from NaCl-induced LL-TSLP cultures or **c** heat-shock-induced LL-TSLP cultures. **d** Detection of IL-12 by ELISA in LPS-induced-BMDCs supernatants co-incubated with commercial recombinant TSLP (rTSLP) (0 or 10 ng/mL), recombinant TSLP produced by LL-TSLP cultures (concentrated TSLP) (10 ng/mL) or with equivalent amount of protein from concentrated supernatant LL-NUC cultures (negative control). Statistically significant differences (*P < 0.05, ***P < 0.001)

In order to study the role of TSLP in mucosal inflammation we constructed a strain of *L. lactis* secreting TSLP (LL-TSLP). To this end, we cloned the *tslp* gene in the plasmid pLB333 carrying the SICE system, composed of the promoter of the stress-induced GroESL operon, the signal peptide of the well secreted *L. lactis* Exp4 protein and a terminator (Fig. 1a), resulting in the plasmid pGroel-TSLP. The LL-TSLP strain had the same growth curve in rich culture medium as the wild type strain MG1363, LL-WT (data not shown). The ability of LL-TSLP to produce the cytokine was tested in different stress conditions such as heat shock and salt stress. We observed a significant (P < 0.001) 35 and 84 % increase of TSLP secretion in presence of 1.5 % NaCl and 3 % NaCl, respectively (Fig. 1b). TSLP secretion is weakly but significantly (P < 0.05) enhanced by heat shock at 37 and 40 °C (Fig. 1c). Finally, to validate the recombinant strain, we tested the biological activity of the secreted TSLP in a LPS-stimulated-BMDCs model. After 24 h of LPS stimulation, we detected IL-12 secretion in BMDCs supernatants, which significantly decreased when cells received TSLP (either commercial recombinant or concentrated from LL-TSLP supernatant), demonstrating a biological activity of the recombinant cytokine (Fig. 1d). Equivalent amount of irrelevant protein also lowers IL-12 secretion, even though significantly different from concentrated LL-TSLP addition. We thus validated the secretion of a biologically active TSLP cytokine by a recombinant *L. lactis* strain.

### Oral administration of LL-TSLP induced TGF-β secretion by activated cells from mesenteric lymph node of healthy mice

**Fig. 2:**
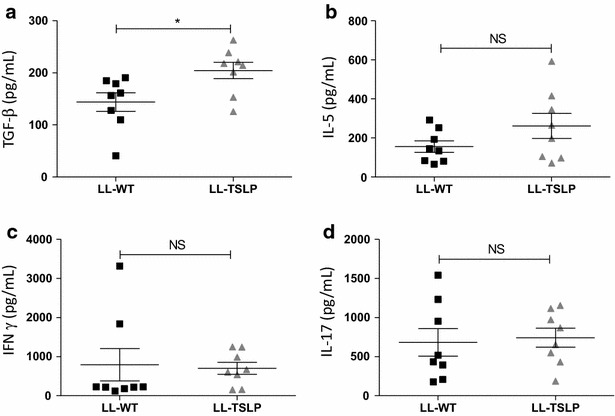
Oral administration of LL-TSLP induced TGF β secretion. Mice were orally administered during 5 days consecutively with LL-TSLP or LL-wt during 5 days consecutively and then sacrificed. Concentrations of TGF-β (**a**), IL-5 (**b**), IFN-γ (**c**) and IL-17 (**d**) were measured in supernatants of anti-CD3 and anti-CD-28 activated cells from MLN

To assess the basal effects of gut mucosal administration of TSLP on mice, two groups (n = 8) of healthy animals received LL-WT, or LL-TSLP by oral route. Weight and DAI were daily monitored and scored. We did not observe differences in these scores, showing no changes in the physiology of mice (data not shown). After 14 days of treatment, mesenteric lymph nodes (MLN) were removed and cells were activated with anti-CD3 and anti-CD-28 antibodies. We detected a significantly (P < 0.05) higher secretion of TGF-β when mice received LL-TSLP compare to mice orally dosed with LL-WT (Fig. 2a). We did not observe any significant changes in IL-5, IFN-γ or IL-17 concentrations in cell supernatants (Fig. 2b–d). No differences have been seen on IL-10 either (data not shown). TSLP delivery through recombinant *L. lactis* in the intestinal lumen is able to trigger TGF-β secretion.

### LL-TSLP reduce acute inflammation

**Fig. 3:**
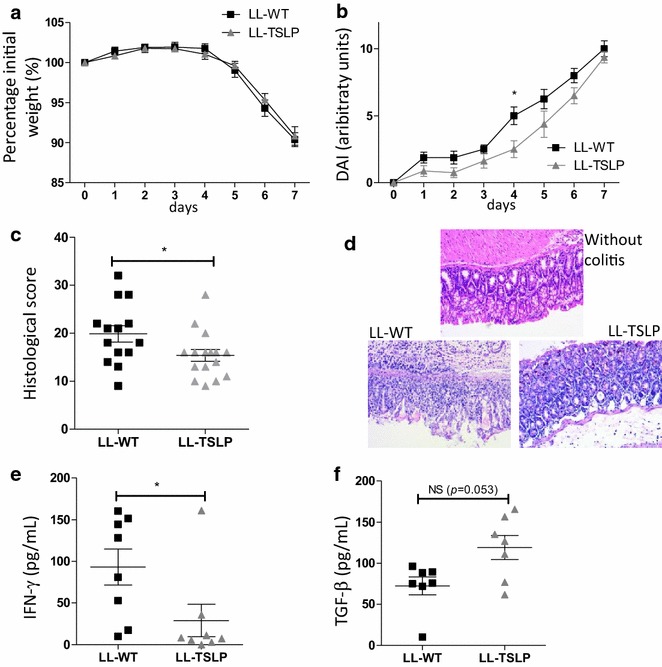
Effects of oral administration of LL-TSLP on acute DSS-induced inflammation in mice. Mice were orally dosed with LL-WT or LL-TSLP 5 days prior DSS treatment until the end of study. Percentage initial weight (**a**) and Disease Activity Index (**b**) were monitored during the whole DSS treatment period. **c** Histological score of colon segment, **d** colon histology, **e** IFN-γ in colon washes and **f** TGF-β concentrations in supernatants of activated cells from MLN

To determine the impact of local administration on intestinal inflammation, we first performed an acute DSS-induced colitis model on mice that we orally administered with LL-TSLP or LL-WT 7 days before and during colitis induction. We did not observe a difference in the weight loss of the two groups of mice (Fig. 3a). Oral administration of LL-TSLP significantly decreased the DAI at D4, showing that TSLP-secreted *L. lactis* delayed clinical signs of colitis (Fig. 3b), especially feces softening and bleeding. After 7 days of inflammation, colon tissues were removed and several inflammation markers were analyzed. Histological score was reduced in presence of TSLP (Fig. 3c, d) demonstrating an intestinal epithelial protection by oral administration of LL-TSLP. The concentration of the pro-inflammatory cytokine IFN-γ in colon washes was also decreased after oral treatment with LL-TSLP (Fig. 3e). We did not detect any differences in the concentration of the pro-inflammatory IL-12 and the anti-inflammatory IL-10 in these colon washes (data not shown). We also observed an increase but not significant (p = 0.053) of TGF-β in the supernatant of activated cells from MLNs. No differences were detected in IL-5, IL-17 or IL-22 concentrations in the supernatant of activated cells from MLNs between the two conditions (data not shown).

### LL-TSLP decreased Disease Activity Index but not weight loss in a DSS recovery phase model

**Fig. 4:**
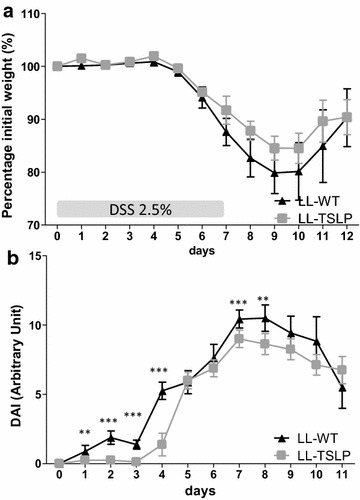
Effects of oral administration of LL-TSLP on a DSS-recovery colitis model in mice. Mice were orally dosed with LL-WT or LL-TSLP 5 days prior DSS treatment until the end of study. After DSS treatment mice were allowed to recover for 5 days. Percentage initial weight (**a**) and DAI (**b**) were monitored during the DSS-induced colitis phase as well as during the following 5 days of recovery

In order to test the involvement of TSLP in the healing process, we performed an acute inflammation experiment followed by a recovery phase consisting of 5 days of water. Two groups of mice were treated 7 days before colitis, along the inflammation as well as the recovery period with LL-WT or LL-TSLP. Oral TSLP administration did not modify the weight loss, which was around a maximum of 20 %, between the two group of mice (Fig. 4a) but LL-TSLP significantly decreased the Disease Activity Index (DAI) at the early phase of the inflammation (Fig. 4b), as seen previously, suggesting that TSLP had no effect in late inflammation and recovery phase.

### LL-TSLP delivery in the early phase of inflammation diminished the loss weight and the DAI

**Fig. 5:**
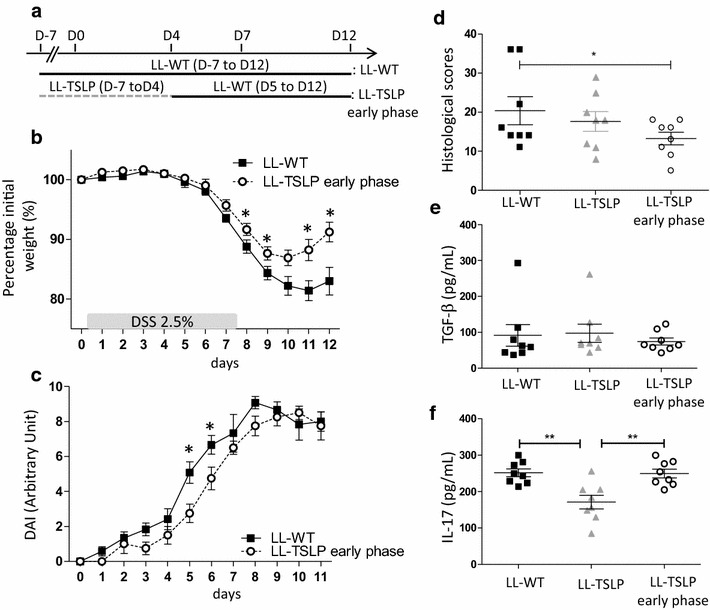
Short and early LL-TSLP administration reduced inflammation during DSS-induced colitis. **a** Schematic representation of bacterial administration protocol. **b** Percentage initial weight. **c** DAI of mice treated with LL-WT or LL TSLP early phase. **d** Histological score. **e** TGF-β and **f** IL-17 concentrations in supernatants of anti-CD3 and anti-CD-28 activated cells from MLN

To validate the effect of TSLP on the early phase of colitis, we performed an acute inflammation followed by a recovery phase on groups of mice treated with LL-WT, LL-TSLP and a group named LL-TSLP early phase, corresponding to an oral administration of LL-TSLP from D7 to D4 followed by oral administration of LL-WT from D5 to D12 (Fig. 5a). As previously shown, the difference in weight loss between LL-TSLP and LL-WT conditions was not significant (data not shown). We observed a reduction of the weight loss when mice received early TSLP delivery, which was significantly different at D8, D9, D11 and D12 compared to the LL-WT condition (Fig. 5b). Furthermore we observed a reduced increase of DAI in the LL-TSLP early phase group, with significant differences at D5 and D6 compared to the LL-WT DAI (Fig. 5c). Histological scores were significantly reduced in the LL-TSLP early phase group compared to the LL-WT group but not in the LL-TSLP group (Fig. 5d). At D12, cells from MLN were activated but we did not detect any differences in TGF-β secretion in these cell supernatants between the three bacterial treatments (Fig. 5e). However, we did notice a significant (P < 0.01) decrease of IL-17 secretion with LL-TSLP administration compare to LL-WT or LL-TSLP early phase (Fig. 5f). These results demonstrated a decrease/amelioration of some colitis symptoms even when TSLP was delivered at the early phase of the inflammation.

### TSLP induce a Treg proliferation in the early phase of the colitis

**Fig. 6:**
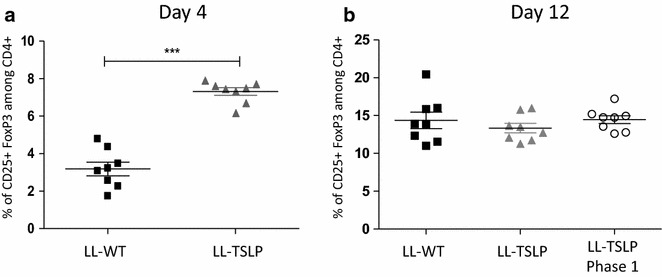
Action of LL-TSLP after 4 days of colitis. Percentage of CD25+FoxP3+ among CD4+ population in MLN at D4 (**a**) and D12 (**b**)

In order to understand the effect of TSLP on the early phase of colitis we analyzed the Treg proportion in MLN at day 4 and day 12 of colitis. The percentage of CD25+ FoxP3 Treg among the CD4+ population was significantly higher when mice were fed with LL-TSLP compared to control LL-WT at day 4 (Fig. 6a). This difference in line with the differences in DAI scores observed between LL-TSLP treated mice and LL-WT at D4 (data not shown). No difference in percentage of CD25+ FoxP3 Treg among CD4+ population was observed at day 12 among the three groups (Fig. 6b).

## Discussion

### In this study, effects of gut mucosal administration of TSLP in treatment for colitis have been investigated using the recombinant *L. lactis* strain LL-TSLP

Three main studies have shown an important role of TSLP in DSS-induced colitis using two knock-out mouse models, where TSLP or the TSLP receptor (TSLPR) gene was deleted. Taylor et al. demonstrated that TSLPR−/− mice were more sensitive to DSS-induced colitis compared to the WT animals [2]. TSLPR−/− mice displayed higher pathological score, from the first day of DSS treatment, and colonic shortening. These macroscopic criteria were correlated with an increase of IFN-γ producing cells within MLNs and IL-12/23p40 secretion in colonic tissue. In contrast, Reardon et al. have shown that the lack of TSLP does not lead to an enhancement of the colitis severity in a DSS model but to an absence of recovery after the inflammation, leading to the death of the mice [27]. During colitis, immune cells are recruited including neutrophils that release high amount of neutrophil elastase (NE). A balance between proteolytic enzymes and their inhibitors is very important in the recovery phase. They observed that TSLP−/− mice have increased NE activity and in parallel, display a diminution of the secretory leukocyte peptidase inhibitor (SLPI), an endogenous inhibitor of NE. Moreover, mortality rate in DSS TSLP−/− mice was reduced by an intraperitoneal rSLPI treatment. The third study has also demonstrated a protective role of TSLP during DSS-induced colitis followed by a recovery phase [13]. In a same manner, the lack of TSLP receptor increased the severity of the colitis monitored by a rise of weight loss, DAI and histological scores. Moreover, TSLPR−/− had higher percentages of Th17 cells and Th1 cells in colonic tissue. Although there are differences in the hypotheses and explanations, all these data show an important role of TSLP in the protection of epithelial integrity and colitis reduction.

In order to further understand the potential protective effect of TSLP on inflammation, we have developed a strategy for TSLP delivery to the gut mucosal level by oral administration of LAB producing soluble functional TSLP. LAB have been used since many years now as vector for protein or DNA delivery at the mucosal level [25]. We constructed and characterized a *L. lactis* strain producing TSLP, LL-TSLP. After 2 weeks of LL-TSLP oral administration in healthy mice we observed an increase of TGF-β production by anti-CD3/anti-CD28 stimulated cells from mesenteric lymph nodes. In an acute DSS-induced inflammation model, we showed that after 7 days of DSS, the DAI of mice treated with LL-TSLP tends to be lower during the 7 days of inflammation, despite absence of changes in weight loss. We observed a significant reduction of this score at D4, demonstrating the capacity of LL-TSLP to delay clinical signs at the beginning of colitis, especially feces softening and bleeding. Furthermore we showed that colonic tissue integrity measured by histological scores is less compromised within TSLP treated mice. Oral administration of LL-TSLP reduced the secretion of the pro-inflammatory cytokine, IFN-γ, showing that LAB-secreted TSLP protects the intestinal epithelium from damages induced by chemical treatment and modulates inflammation.

To assess the effect of LL-TSLP during the recovery phase, we performed an acute colitis model followed by 5 days of remission. TSLP was delivered by LL-TSLP all along the experiment (inflammation + recovery phase). We did not show any differences in weight loss and histological scores after 5 days of water but we confirmed the decrease of DAI in the early phase of inflammation. The recovery phase is a complex process and addition of TSLP seems not to be sufficient to accelerate the decrease of markers of inflammation or intestinal epithelium repair.

Next, we hypothesized that early treatment with LL-TSLP could be sufficient to decrease inflammation markers. A group of mice received LL-TSLP during 7 days before and 4 days after the induction of colitis followed by LL-WT until the end of the experiment. TSLP delivery in the lumen at early phase, until D4, diminished the weight loss and significantly increased the weight gain at D8, D9, D11 and D12 compare to the LL-WT. Moreover it delayed and decreased the DAI (significantly at D5 and D6) and reduced the histological score. Therefore we conclude that, short and early TSLP treatment allowed a better protection against colitis than a longer treatment as demonstrated by a lower severity as well as a delay in the disease.

To decipher by which mechanisms addition of TSLP leads to the colon protection in the early phase of the inflammation, we sacrificed the mice at D4. At this time we observed a higher percentage of CD4+CD25+Foxp3+ cells in mice treated with LL-TSLP, suggesting a role of Treg cells in the delay of the outbreak of the disease. In human, TSLP-matured DC are able to induce the expansion and the differentiation of CD4+CD25+Foxp3+ cells [14, 15]. However, Iliev et al. have demonstrated that the retinoic acid and TGF-β but not TSLP are essential to convert DC in tolerogenic DC, which induce the Treg differentiation [28] and lead to the protection of mice from colitis. In parallel, TSLP has been described to be secreted by DC [13, 29]. DC-derived TSLP can directly act on Tcell by inhibiting TH17 cell development and promoting Treg cells [13]. TSLP was also described to be essential for gut homeostasis and TSLP-TSLPR signaling elicits the expansion of Foxp3+ helios-Treg cells in response to intestinal bacteria [30].

We hypothesized that addition of TSLP to the lumen allows an enhancement of gut homeostasis by a rise of the number of Treg cells which leads to a delay of the disease. Release of TSLP could act directly as described by Spadoni et al. on Treg differentiation or indirectly. Indeed, TSLP is able to reinforce tight-junctions of lung epithelial cells by increasing several claudins and the occludin [31]. In this manner, TSLP could protect gut epithelial integrity and increase the release of Retinoic acid and TGF-β by epithelial cells as well as the Treg expansion.

Finally, TSLP expression is reduced in colonic tissue of Crohn’s disease patients [32] and can be correlated to the failure of these patients to promote tolerogenic DCs in the gut [4, 28]. TSLP secretion by intestinal epithelial cells is dependant and regulated by commensal and probiotic bacteria [30, 33, 34]. A novel treatment against Crohn disease is fecal transplantation. In the future, it could be very interesting to target fecal transplant that restore TSLP expression or complete actual treatment with probiotics that are able to increase TSLP secretion by epithelial cells to promote gut homeostasis and longer remission periods.

## Conclusion

Thymic stromal lymphopoietin showed an anti-inflammatory protective role in DSS-induced colitis. We have demonstrated that a short and early administration of LL-TSLP is more efficient than a long lasting treatment. We hypothesized that TSLP induced TGF-β secretion which thus will increase the Treg population.

## Methods

### Bacterial strains and growth conditions

*Lactococcus lactis* MG1363 containing pNis-empty plasmid (LL-WT) and *L. lactis* MG1363 containing pGroESL-TSLP plasmid (LL-TSLP) were grown in M17 medium (Difco) supplemented with 1 % glucose and chloramphenicol (10 µg/mL) at 30 °C without agitation. pSEC plasmid and pGroESL are both derivative of the broad-host range plasmid pWV01 [35].

### Plasmid construction

Plasmid containing a murine *tslp* gene was synthesized by Geneart (Invitrogen). After digestion by *Bam*HI/*Spe*I, the fragment containing the gene of interest was integrated into a *Bam*HI/*Spe*I digested pGroESL plasmid (chloramphenicol resistant, gene expressed under P_groESL_, flanking by signal peptide from USP45 and a terminator) from SICE system [36]. Construction was established by electroporation into *L. lactis* MG1363 at 2.4 kV, 200 Ω, 25 µF. Transformants were selected at 30 °C on M17 agar containing 1 % glucose and chloramphenicol (10 µg/mL). pGroESL-TSLP plasmids were extracted from recombinant transformants and verified by sequencing.

### Stress inducing cytokine secretion by *L. lactis*

Overnight cultures of TSLP-secreting *L. lactis* strain was diluted in growth medium to a 0.1 OD_600nm_ and incubate at 30 °C without agitation until a 0.4–0.6 OD_600nm_. Then different stresses were added as following: To induce a salt stress, different volumes of NaCl 5M solution were added into culture to obtain 1, 5 and 3 % NaCl final concentration and incubate at 30 °C without agitation. To provoke a heat-shock, bacterial cultures were centrifuged at room temperature at 4700 rpm during 15 min. Pellets were resuspended with pre-warmed culture medium at 30, 37 and 40 °C and incubate at these different temperatures without agitation. Four hours after stress, 1 mL of bacterial cultures was harvested and centrifuged at 4 °C at 10,000 rpm during 10 min. The 2 µm filtered supernatants were conserved at −20 °C for cytokine quantification by ELISA (R&D systems).

### Isolation and culture of bone marrow-derived dendritic cells (BMDC)

Bone marrow cells from BALB/c mice were harvested aseptically and plated into a petri dish in RPMI 1640 (Life Technologies) supplemented with 10 % decomplemented fetal bovine serum (FBS), penicillin/streptomycin, β-mercaptoethanol 5 mM and 20 ng/mL GMCSF (peprotech) and cultured at 37 °C in a 10 % CO_2_-humidified incubator. Fifteen mL of medium were added at day 3 and completely changed at day 5; cells were harvest at day 7. BMDCs were then plated at 5 × 10^5^ cells/well (96 wells/plate) and cultured in RPMI 1640 supplemented with 10 % decomplemented FBS and penicillin/streptomycin at 37 °C in a 10 % CO_2_-humidified incubator.

### LPS-stimulated-BMDC assay

BMDCs were stimulated in presence (5 ng/mL) or absence of LPS and with recombinant TSLP (Biolegend or from LL-TSLP concentrated supernatants) at 10 ng/mL. A negative control of the culture medium (filtered supernatant of *L. lactis* harboring a plasmid encoding for a non-relevant protein, the nuclease Nuc) was used and equivalent protein amount corresponding to concentrations used with concentrated TSLP was added. Twenty-four hours after stimulation, cells supernatants were harvested for IL-12 quantification by ELISA (mabTech).

### Mice experiments

After acclimatization during at least 7 days, 6 weeks old C57BL/6 mice were fed daily during the whole experiment with PBS or with 10^9^ ~ 5 × 10^9^ Colony Forming Units of LL-WT or LL-TSLP. At D0 colitis was induced by adding 2.5 % (w/v) of Dextran Sulfate Sodium Salt (DSS) at a molecular weight of 36,000–50,000 (MPBio) to the drinking water for 4 days (DSS short) or 7 days (DSS acute and DSS recovery). The mice were sacrificed either at D4 (DSS short), D7 (DSS acute) or D12 (DSS recovery) after the DSS induction. For DSS recovery, DSS colitis induction was followed by 5 days of recovery with normal drinking water. As a control DSS mice have been fed during 12 days without DSS induction.

Mice were monitored daily for weight loss, stool consistency, and fecal occult blood (Hemoccult, Beckman Coulter). Disease Activity Index (DAI) has been calculated according to the protocol established by Cooper et al. [37].

Mice have been sacrificed by cervical dislocation and mesenteric lymphatic node (MLN) as well as colon have been harvested for colon washes, protein extraction and histological assessment.

### Interleukin detection in colon washes, soluble protein extracts from colonic tissues and supernatant from induced lymphocyte

The colon was removed and washed with 1 mL of PBS supplemented with anti-protease to obtain the colon wash.

Then the colon was opened and 1 cm was removed. The slice was grinded in 1 mL of PBS supplemented with anti-protease using a GentleMacs (Miltenyi) then centrifuged 2 min at 5000*g*. Supernatant was stored at −20 °C before further analysis.

MLN isolated from mice were mashed and filtered (70 µm, BD biosciences). Lymphocytes in filtrate were count by flow cytometry (Accuri C6) and resuspended in culture medium (RPMI, Lonza) with 100 Unit of Streptomicin Penicilin, PAA Laboratories and 10 % Fetal Calf Serum (FCS) (Lonza) at 25 × 10^6^ cells/mL. Cell solutions were added to 24 well plates (Costar) pre-incubated 4 h with anti-CD3 and anti-CD28 antibodies, 4 µg/mL of each antibody (eBioscience) in PBS with 0.5 % FCS. Plates were incubated 48 h at 37 °C 5 % of CO_2_ and cytokine levels were assessed in supernatant.

Total amount of proteins from the different samples was determined using Bradford assay (Sigma-Aldrich) and the cytokine concentration (IL-10, IL-12, IFN-γ, TGF-β, IL-17, IL-22 and IL-5) by ELISA (Mabtech). The concentration of each cytokine is normalized on the total proteins amount of each samples.

### Histological assessment

For histological assessment, a colon sample was fixed in 4 % paraformaldehyde acid (sigma) and embedded in paraffin. Four micrometer sections were stained with hematoxylin/eosin and examined blindly [12].

### Regulatory T cells (Treg) numeration

10^6^ cells have been taken from mashed MLN filtrates. Treg cells have been stained for CD4, CD32 and FoxP3 using a mouse regulatory T cell Staining Kit 1 (eBioscience). Cell samples have been run through flow cytometry (BD Accuri) and double positive cells for CD32 and FoxP3 among CD4 positive cells have been counted.

### Statistic

All statistics and graphics have been performed on Prism-GraphPad^®^. Results represent mean ± SEM. Statistical significance was determined by the Mann–Withney test for charts and by 2-way Anova with Bonferroni post-test for curves *P < 0.05, **P < 0.01, **P < 0.001.

## Ethical statement

All animal assays were performed following the European Guidelines for the Care and Use of Laboratory Animals. Authorization number: 02550.01.

## References

[1] Friend S, Hosie S, Nelson A (1994). A thymic stromal cell line supports in vitro development of surface IgM+ B cells and produces a novel growth factor affecting B and T lineage cells. Exp Hematol.

[2] Taylor BC, Zaph C, Troy AE (2009). TSLP regulates intestinal immunity and inflammation in mouse models of helminth infection and colitis. J Exp Med.

[3] Zaph C, Troy AE, Taylor BC (2007). Epithelial-cell-intrinsic IKK-beta expression regulates intestinal immune homeostasis. Nature.

[4] Rimoldi M, Chieppa M, Salucci V (2005). Intestinal immune homeostasis is regulated by the crosstalk between epithelial cells and dendritic cells. Nat Immunol.

[5] Ziegler S, Artis D (2010). Sensing the outside world: TSLP regulates barrier immunity. Nat Immunol.

[6] Lee H, Ziegler SF (2007). Inducible expression of the proallergic cytokine thymic stromal lymphopoietin in airway epithelial cells is controlled by NFkappaB. Proc Natl Acad Sci USA.

[7] Ying S, O’Connor B, Ratoff J (2005). Thymic stromal lymphopoietin expression is increased in asthmatic airways and correlates with expression of Th2-attracting chemokines and disease severity. J Immunol..

[8] Blázquez AB, Mayer L, Berin MC (2010). Thymic stromal lymphopoietin is required for gastrointestinal allergy but not oral tolerance. Gastroenterology.

[9] Mou Z, Xia J, Tan Y (2009). Overexpression of thymic stromal lymphopoietin in allergic rhinitis. Acta Otolaryngol.

[10] Pedroza-Gonzalez A, Xu K, Wu T-C (2011). Thymic stromal lymphopoietin fosters human breast tumor growth by promoting type 2 inflammation. J Exp Med.

[11] De Monte L, Reni M, Tassi E (2011). Intratumor T helper type 2 cell infiltrate correlates with cancer-associated fibroblast thymic stromal lymphopoietin production and reduced survival in pancreatic cancer. J Exp Med.

[12] Dieleman LA, Palmen MJHJ, Akol H (1998). Chronic experimental colitis induced by dextran sulphate sodium (DSS) is characterized by Th1 and Th2 cytokines. Clin Exp Immunol..

[13] Spadoni I, Iliev ID, Rossi G (2012). Dendritic cells produce TSLP that limits the differentiation of Th17 cells, fosters Treg development, and protects against colitis. Mucosal Immunol.

[14] Hanabuchi S, Ito T, Park WR (2010). Thymic stromal lymphopoietin-activated plasmacytoid dendritic cells induce the generation of FOXP3+ regulatory T cells in human thymus. J Immunol.

[15] Watanabe N, Wang Y-H, Lee HK (2005). Hassall’s corpuscles instruct dendritic cells to induce CD4+CD25+ regulatory T cells in human thymus. Nature.

[16] Lakatos P (2006). Recent trends in the epidemiology of inflammatory bowel diseases: up or down?. World J Gastroenterol.

[17] Molodecky NA, Soon IS, Rabi DM (2012). Increasing incidence and prevalence of the inflammatory bowel diseases with time, based on systematic review. Gastroenterology..

[18] Lovasz BD, Golovics PA, Vegh Z (2013). New trends in inflammatory bowel disease epidemiology and disease course in Eastern Europe. Dig Liver Dis..

[19] Lehner T, Challacombe SJ, Caldwell J (1976). Immunologic basis for vaccination against dental caries in rhesus monkeys. J Dent Res.

[20] Arvikar SL, Fisher MC (2011). Inflammatory bowel disease associated arthropathy. Curr Rev Musculoskelet Med..

[21] Huang BL, Chandra S, Shih DQ (2012). Skin manifestations of inflammatory bowel disease. Front. Physiol..

[22] Macdougall I (1964). The cancer risk in ulcerative colitis. Lancet.

[23] Guerra I, Bermejo F (2014). Management of inflammatory bowel disease in poor responders to infliximab. Clin Exp Gastroenterol..

[24] Motta J-P, Bermúdez-Humarán LG, Deraison C (2012). Food-grade bacteria expressing elafin protect against inflammation and restore colon homeostasis. Sci Transl Med..

[25] Bermúdez-Humarán LG, Aubry C, Motta J-P (2013). Engineering lactococci and lactobacilli for human health. Curr Opin Microbiol.

[26] Chen L-L, Zou Y-Y, Lu F-G (2013). Efficacy profiles for different concentrations of *Lactobacillus acidophilus* in experimental colitis. World J Gastroenterol.

[27] Reardon C, Lechmann M, Brüstle A (2012). Thymic stromal lymphopoetin-induced expression of the endogenous inhibitory enzyme SLPI mediates recovery from colonic inflammation. Immunity.

[28] Iliev ID, Mileti E, Matteoli G (2009). Intestinal epithelial cells promote colitis-protective regulatory T-cell differentiation through dendritic cell conditioning. Mucosal Immunol.

[29] Kashyap M, Rochman Y, Spolski R (2011). Thymic stromal lymphopoietin is produced by dendritic cells. J Immunol..

[30] Mosconi I, Geuking MB, Zaiss MM (2013). Intestinal bacteria induce TSLP to promote mutualistic T-cell responses. Mucosal Immunol.

[31] Kamekura R, Kojima T, Koizumi J (2009). Thymic stromal lymphopoietin enhances tight-junction barrier function of human nasal epithelial cells. Cell Tissue Res.

[32] Noble CL, Abbas AR, Lees CW (2010). Characterization of intestinal gene expression profiles in Crohn’s disease by genome-wide microarray analysis. Inflamm Bowel Dis.

[33] Mileti E, Matteoli G, Iliev ID (2009). Comparison of the immunomodulatory properties of three probiotic strains of lactobacilli using complex culture systems: prediction for in vivo efficacy. PLoS One.

[34] Zeuthen LH, Fink LN, Frokiaer H (2008). Epithelial cells prime the immune response to an array of gut-derived commensals towards a tolerogenic phenotype through distinct actions of thymic stromal lymphopoietin and transforming growth factor-β. Immunology.

[35] Kok J, van der Vossen JM, Venema G (1984). Construction of plasmid cloning vectors for lactic streptococci which also replicate in *Bacillus subtilis* and *Escherichia coli*. Appl Environ Microbiol.

[36] Benbouziane B, Ribelles P, Aubry C (2013). Development of a Stress-Inducible Controlled Expression (SICE) system in *Lactococcus lactis* for the production and delivery of therapeutic molecules at mucosal surfaces. J Biotechnol.

[37] Cooper HS, Murphy SN, Shah RS (1993). Clinicopathologic study of dextran sulfate sodium experimental murine colitis. Lab Investig..

