# Erratum to: Protective effect of TSLP delivered at the gut mucosa level by recombinant lactic acid bacteria in DSS-induced colitis mouse model

**DOI:** 10.1186/s12934-016-0592-6

**Published:** 2016-11-10

**Authors:** Camille Aubry, Christophe Michon, Florian Chain, Yolande Chvatchko, Laurence Goffin, Simone C. Zimmerli, Sylvia Leguin, Philippe Langella, Luis Bermudez-Humaran, Jean-Marc Chatel

**Affiliations:** 1 INRA, UMR1319 Micalis, 78350 Jouy-en-Josas, France; 2 AgroParisTech, UMR Micalis, 78350 Jouy-en-Josas, France; 3 Merck Serono SA, 1170 Aubonne, Switzerland; 4 EMD Serono, 45A Middlesex Turnpike, Billerica, MA 01821 USA

## Erratum to: Microb Cell Fact (2015) 14:176 DOI 10.1186/s12934-015-0367-5

After publication of the original article [1] it was brought to our attention that author Yolande Chvatchko was incorrectly included as Yolande Chvatchenko. The correct spelling of the name is included in the author list of this erratum.
